# Are Informing Knowledge and Supportive Attitude Enough for Tobacco Control? A Latent Class Analysis of Cigarette Smoking Patterns among Medical Teachers in China

**DOI:** 10.3390/ijerph121012030

**Published:** 2015-09-25

**Authors:** Lu Niu, Dan Luo, Vincent M.B. Silenzio, Shuiyuan Xiao, Yongquan Tian

**Affiliations:** 1 Department of Social Medicine and Health Management, School of Public Health, Central South University, Changsha 410078, Hunan, China; E-Mails: angela_niulu@hotmail.com (L.N.); xiaosy@csu.edu.cn (S.X.); tianyq@csu.edu.cn (Y.T.); 2 Department of Psychiatry, University of Rochester Medical Center, Rochester, New York, NY 14642, USA; E-Mail: vincent_silenzio@urmc.rochester.edu

**Keywords:** smoking, smoking cessation, health knowledge, attitude, practice

## Abstract

*Background*: This study is one part of a five-year tobacco-control project in China, which aimed to gain insight into the smoking behavior, knowledge, and attitudes among medical teachers in China. *Methods*: In May 2010, a cross-sectional survey was conducted among medical teachers of Xiangya Medical School, Central South University, China. *Results*: A total number of 682 medical teachers completed the surveys. Latent class analysis indicated the sample of smoking patterns was best represented by three latent subgroups of smoking consumption severity levels. Most respondents were informed of smoking related knowledge, but lack of knowledge on smoking cessation. Most of them held a supportive attitude towards their responsibilities among tobacco control, as well as the social significance of smoking. However, both smoking related knowledge and attitude were not correlated with severity of smoking consumption among medical teachers. *Conclusion*: The smoking prevalence among medical teachers in China remains high. Programs on smoking cessation training are required. Future study should also develop targeted interventions for subgroups of smokers based on smoking consumption. Persistent and effective anti-tobacco efforts are needed to achieve the goals of creating smoke-free campuses and hospitals.

## 1. Introduction

Tobacco use continues to be the leading global cause of preventable death, while six million people die of tobacco related diseases yearly [1], of whom one million are Chinese [2]. The Chinese government has made many efforts to fight tobacco use in order to curb the burgeoning epidemic, but the smoking prevalence in China is still very high especially among male adolescents and adults. The data from the former Chinese Ministry of Health showed that the smoking prevalence among Chinese population aged 15 years old and above was 28.1%, specifically, 52.9% among male adults [3]. 

The important role of health care providers (HCPs) in tobacco control has been well documented, including role modeling of health behaviors, as well as providers and educators of information related to tobacco cessation [4,5]. In some developed countries, such as Canada, the USA, Sweden, Australia, and the UK, the smoking among HCPs is not a major problem as the prevalence is very low [6]. Meanwhile, considerably high smoking prevalence among Chinese physicians was found, with 56.8% of male doctors [7] using tobacco, which makes smoking in itself a critical issue. Based on the knowledge, attitudes and practice (KAP) model, previous studies suggested that the insufficiency of tobacco-related knowledge and the inadequacy of attitudes regarding smoking cessation might be the key reasons for the high smoking prevalence among HCPs, and pose barriers in tobacco control [8,9]. Thus, education on tobacco control needs to be increased among HCPs. Also medical schools should have tobacco related education as part of the curriculum for medical students—the future HCPs—to protect them from smoking habits in advance [10,11]. 

Teachers in medical schools often wear two hats as HCPs for patients and teachers for medical students. This should be emphasized by their responsibilities in tobacco control and their own smoking status, but we only found two studies which focus on medical teachers in China [12,13] and one in Iran [14]. Specifically, 49.5% of Chinese male medical teachers had smoked more than 100 cigarettes in their lifetime [12], compared with 27.2% of those in Iran [14]. It is imperative to fill the gap in the existing literature, in order to understand the smoking behavior, knowledge and attitudes among medical teachers, and plan for more effective strategies and interventions to curb the smoking issue in China.

*The China Medical Tobacco Control* is a five-year project supported by the China Medical Board, which has been conducted within leading medical universities across China since 2010 [15]. It has aimed to assess the smoking condition among medical school and hospitals, and create smoke-free campuses and hospitals by designing a strong, efficient smoking controlling system. The baseline findings specific to medical teachers among Central South University are presented here. 

## 2. Methods

### 2.1. Setting and Sample

Xianya Medical School of Central South University, located in Changsha city, Hunan province, is one of the leading medical schools in China. It consists of six colleges (medical school, nursing school, public health school, stomatology, pharmaceutical sciences school, and life sciences school) and five affiliated hospitals. In May 2010, a cross-sectional survey was conducted among medical teachers in the six colleges and one of the affiliated hospitals. Questionnaires were distributed and then collected in a one-week period. The research protocols were reviewed and approved by the Institutional Review Board of Central South University, and participants gave written informed consent.

### 2.2. Instruments

#### 2.2.1. Demographic Characteristics

Socio-demographic characteristics included gender (male, female), age (in number of years), ethnicity (Han, minority), educational level (Bachelor, Master, PhD), and married status (single, married, divorced or widowed).

#### 2.2.2. Smoking Behaviors 

Smoking behaviors were measured by the questionnaire of China Medical Tobacco Control Baseline Survey [13,16]. Smoking behaviors include questions on smoking status, initial stage of smoking, smoking setting and quitting, ideation and attempt.

*Smoking status.* Smoking status was measured through four questions: “Have you ever smoked a cigarette, even just taken a puff?” “How many cigarettes have you smoked in your lifetime?” “How many days have you smoked in the last 30 days?” and “How many cigarettes have you smoked on average per day in the last 30 days?” The self-reported smoking statuses were categorized as: (1) ever smoker: an individual who has ever smoked a cigarette, even just taken a puff; (2) current smoker: an individual who has smoked at least one cigarette in the last 30 days [17,18]. We defined “never smokers” as people who have never smoked, while ever smokers were also defined as “smokers”.

*Initial age of smoking.* Smokers were asked about their initial age of smoking their first cigarette.

*Smoking setting.* Current smokers were asked whether they had smoked on campus or in the hospital in the last 30 days. 

*Smoking cessation.* Current smokers were asked about their smoking cessation ideation (“Do you want to quit smoking?”) and attempt (“Have you made quitting attempts in the last 12 months?”).

#### 2.2.3. Attitude towards Tobacco

Attitude toward tobacco was measured with eight items by the questionnaire of the China Medical Tobacco Control Baseline Survey [13,16]. There were five items about whether people could benefit from smoking. Respondents indicated on a three-point Likert type scale, where “0” represented agreement, “1” no opinion, and “2” disagreement. The other three items were about the attitudes toward medical teachers’ responsibilities among smoking control. Response “Agree” scored two, “Disagree” scored zero. Scores of all items could range from 0 to 16. Lower scores indicate a more favorable attitude toward tobacco use.

#### 2.2.4. Russell’s Reasons for Smoking Questionnaire (RRSQ) 

The adapted version of RRSQ instrument [19,20] was used to identify smoking motive factors of current smokers, which consists of 24 items on a four-point scale (0 = not at all, 3 = frequently). It includes seven factors (psychological image, hand-mouth activity, indulgent, sedative, stimulant, addictive, and automatic), and each of them consists of three items. Dependence score was calculated by the sum of scores on addictive, automatic, and three other items (“I get a definite craving to smoke when I have to stop for a while”; “I find it difficult to go as long as an hour without smoking”, “I would find it difficult to go without smoking for as long as a week”). A dependence score >6 indicates that one may be addicted to tobacco using, and a score >20 indicates that one may be heavily addicted. The overall score could range from 0 to 72. The Cronbach’s α was 0.95.

#### 2.2.5. Knowledge towards Tobacco

Smoking related knowledge was measured by the knowledge section of KAP (knowledge, attitude, and practices) Survey for HCPs (Cronbach’s α = 0.84) [9]. This instrument included 30 items about smoking related health hazard (general effects and disease-specific consequence), physiological effects, and tobacco addiction. Each item had three answer options “True”, “False” or “Unknown”. Correct response was scored as one, while wrong or “Unknown” response was scored as zero. The scores for all questions were added together, and the total score of smoking-related knowledge could range from 0 to 30.

### 2.3. Statistical Analysis

SPSS (version 17.0) was utilized to calculate proportions, ranges (minimum and maximum), means and standard deviations, as well as median and interquartile rang (IQR) of descriptive variables. Chi-squared tests, Mann-Whitney U tests and Kruskal-Wallis H tests were used for comparison among different groups. Spearman correlation analysis was used to evaluate the relationship between scores of RRSQ and the average number of cigarette and days smoked in the past 30 days of current smokers.

Latent class analysis (LCA) was used to identify homogeneous groups with similar smoking patterns. Beginning with an unconditional model (*i.e.*, one-class model), we compared the model fit indictors for an increasing number of classes, and selected the model that provided the best fit to the data. The entropy, Akaike Information Criterion (AIC), Bayesian Information Criterion (BIC), sample size adjusted Bayesian Information Criterion (SSABIC), and Lo-Mendell-Rubin adjusted likelihood ratio test (LRT) were used as tools to guide the determination of how well the different number of classes separated the different smoking patterns. Mplus (version7.2) was utilized. Then, multinomial regression (conducted in SPSS) was used to explore the significant predictors of latent class membership. For all analyses, a significance level of *p* ≤ 0.05 was used.

## 3. Results

### 3.1. Sample Characteristics

We had distributed a total of 869 questionnaires, and a total number of 682 medical teachers completed the surveys, giving a valid response rate of 78.5%. There were 351 (51.5%) males and 331 (48.5%) females, with mean age of 37.0 ± 8.3 years. The majority of the participants were Han (91.1%), married (76.5%), had at least Master degree (70.4%), and were HCPs (55.9%). 

### 3.2. Smoking Prevalence

Among 682 respondents, 48.7% (332) reported that they had ever smoked a cigarette, even just taken a puff (male 77.5%; female 18.1%; *p* < 0.01), while 26.0% (177) had smoked in the last 30 days (male 45.6%; female 2.4%; *p* < 0.01). Most smokers (58.0%) had their first cigarette after being 18 years old (attending college). The initial age of smoking was positively correlated with the age of smokers (r = 0.307, *p* = 0.000).

Almost half the current smokers (46.3%) smoked over 20 days during the past 30 days and one-third (30.5%) consumed at least half a pack of cigarettes per day. The majority of current smokers (59.0%) reported smoking in campus or hospital, while 68.9% stated that if someone offered a cigarette to them, and asked them to smoke together with them, they would be very pleased to accept it. The mean score of RRSQ of current smokers was 13.5 ± 12.4, with a range of 0 to 72, while 27.5% had a dependence score >6. The most common smoking motive factors were addiction (median 2, IQR 0–7) and sedative (median 2, IQR 1–4). The RRSQ scores were positively correlated with the smoking frequency (r = 0.513, *p* = 0.000) and average cigarette consumed per day (r = 0.307, *p* = 0.000) in the last 30 days.

In addition, 51.8% of current smokers reported the will to quit smoking, and 42.3% had made an attempt in the past 12 months but failed.

### 3.3. Latent Class Analysis

Three parameters were used to identify homogeneous groups with similar smoking patterns: (1) quantity of cigarettes consumed in lifetime; (2) frequency of consumption in the last 30 days; and (3) quantity of cigarettes consumed per day in the last 30 days (Table 1). Fit indices for the latent class analysis are shown in Table 1. The three-class model was the best fitting model for the data, as it had the lowest AIC, BIC, and SSABIC, as well as having an excellent entropy value (0.967).

Overall, the three-class model represented the continuum of levels of smoking consumption severity (Table 2). The majority (74.1%) of participants were assigned to Class I, a group with non-smokers and those who had ever smoked but not in the last 30 days; 12.3% was assigned to Class II, a group who smoked with moderate number of cigarettes consumed and frequency of smoking in the last 30 days; and 13.6% were identified in Class III, a group with higher level of smoking consumption and frequency. On further analysis, we found Class III had a significantly higher dependence score compared to Class II (6.6 ± 5.6 *vs*. 2.0 ± 3.4; *p* = 0.000). There was no difference of smoking cessation ideation (45.2% *vs.* 58.3%; *p* = 0.054) and attempts (38.7% *vs.* 48.8%, *p* = 0.215) among Class II and Class III.

**Table 1:** Fit indices for the latent class analysis of smoking behaviors.

Fit Indices	Model				
	Two-Class	Three-Class	Four-Class	Five-Class	Six-Class
Log likelihood	−1115.551	−1056.873	−1054.381	−1054.294	−1054.294
Free parameters	13	20	27	34	41
AIC	2257.102	2153.747	2162.762	2176.588	2190.588
BIC	2315.928	2244.247	2284.938	2330.439	2376.114
SSABIC	2274.651	2180.745	2199.210	2222.484	2245.934
LRT *p* value	0.000	0.000	0.021	0.631	0.183
Entropy	0.998	0.967	0.955	0.943	0.587

Notes: AIC: Akaike information criteria; BIC: Bayesian information criteria; SSABIC: Sample size adjusted Bayesian information criteria; LRT: Lo-Mendell-Rubin adjusted likelihood ratio test.

**Table 2:** Three-class item-response probabilities of smoking behavior measures.

Smoking Behavior Indicators	Class I (*n* = 505, 74.1%)	Class II (*n* = 84, 12.3%)	Class III (*n* = 93, 13.6%)
Quantity in lifetime ^a^			
0	0.701	0.000	0.000
1–99	0.264	0.584	0.000
≥100	0.035	0.416	1.000
Frequency (day) ^b^			
0	1.000	0.064	0.041
1–19	0.000	0.861	0.115
20–30	0.000	0.075	0.844
Quantity per day ^c^			
0	1.000	0.012	0.000
1–10	0.000	0.988	0.367
≥11	0.000	0.000	0.633

**^a^** Quantity of cigarettes consumed in lifetime; **^b^** Frequency of consumption in the last 30 days; **^c^** Quantity of cigarettes consumed per day in the last 30 days.

### 3.4. Smoking Related Knowledge

Among the 577 respondents (84.6%) who completed the whole knowledge items, the mean score of smoking related knowledge is 22.1 ± 4.6, with a range of 0 to 30. Most respondents knew about the harmful effects of smoking and of second-hand smoking on the respiratory system and pregnancy. Notably, however, about half the respondents believed that “low-tar and low-nicotine cigarettes can reduce the harmful effects of smoking on health” (53.0%); one-third agreed that “filers can make smoking safe” (30.5%), and “it is too late to benefit from quitting smoking if you are more than 40 years old” (30.3%). Only 39.1% of respondents were informed that smoking cessation was difficult. Females had slightly higher knowledge scores than males (22.8 ± 4.0 *vs*. 21.5 ± 5.0; *p* = 0.008). There was also significant difference between three latent classes (*p* = 0.002). Especially, Class I was more likely to have high knowledge scores than the other two groups (*p* < 0.05). There was also no association between knowledge score and smoking quitting ideation among current smokers (*p* = 0.986).

### 3.5. Attitude towards Smoking 

Among 621 respondents (91.1%) who completed the attitude toward smoking items, the mean score is 8.6 ± 3.0 (male, 8.4 ± 3.2; female, 8.9 ± 2.8, *p* = 0.006), with a range from 0 to 16. More than half of the respondents agreed that smokers could benefit from smoking by relieving pressure and anxiety, improving social communication, and feeling excited and happy (76.8%, 62.7%, and 55.0%, respectively); and a majority of respondents agreed that medical teachers should play a leading role on tobacco control (92.9%); medical teachers are obligated to refuse smoking (86.0%); and medical teachers have the responsibilities to exhort the public to quit smoking (82.5%). Participants in Class III had significantly lower attitude scores than those in Class I (7.9 ± 3.2 *vs*. 8.9 ± 3.0, *p* = 0.009). Specifically, people with higher smoking consumption behaviors tended to agree that smoking could help them concentrate and improve social communication; but were less likely to agree about the responsibilities of medical teachers to refuse smoking and exhort the public to quit smoking (*p* < 0.05). There was also no association between attitude score and smoking quitting ideation (*p* = 0.514).

**Table 3:** Multinomial regression analysis results.

Variables	Class II	Class III
Gender (male)	38.56 (11.78–126.19) *	57.27 (13.56–241.85) *
Age ^a^		
31–40	1.47 (0.60–3.58)	8.08 (2.03–32.18) *
>40	1.29 (0.48–3.49)	8.35 (2.01–34.74) *
Ethnicity ^b^		
Minority	2.14 (0.74–6.23)	1.36 (0.40–4.64)
Educational background ^c^		
Master	1.10 (0.51–2.38)	0.55 (0.25–1.20)
PhD	0.96 (0.42–2.18)	0.54 (0.24–1.19)
Married status ^d^		
Married	0.98 (0.41–2.36)	1.83 (0.53–6.33)
Divorced or widowed	1.19 (0.17–8.27)	3.47 (0.53–22.59)
Position ^e^		
Non–HCPs	1.09 (0.56–2.12)	0.71 (0.37–1.39)
Knowledge ^f^		
≥23	1.19 (0.66–2.13)	1.35 (0.72–2.51)
Attitude ^g^		
≥9	1.22 (0.68–2.19)	1.68 (0.90–3.15)

**^a^** Reference group = Aged 23 to 30 years; **^b^** Reference group = Han; **^c^** Reference group = Bachelor degree; **^d^** Reference group = Single; **^e^** Reference group = HCPs; **^f^** Reference group = Knowledge score <23; **^g^** Reference group = Attitude score <9; * *p* <0.01.

### 3.6. Multinomial Regression Analysis

Table 3 presents the results of multinomial regression, with Class I treated as the reference group, and gender as covariate factor. Compared to Class I, Class III were more likely to be older (aged above 30 years). Both smoking related knowledge and attitude were not correlated with severity of smoking consumption.

## 4. Discussion

This present study was conducted in one medical school, in which the smoking status is typical of that in China’s medical schools, and could reflect the common situations of Chinese medical teachers. We found that the prevalence of current smoking among medical teachers was 26.0%, and specifically, almost half (45.6%) male were current smokers, which was about the same as in the Chinese general population [21]. It was also consistent with a national survey among college teachers, in which, the prevalence of current smoking was 27.9% (41.9% in male) [22]. Additionally, medical faculty with health care duties reported comparable smoking prevalence with those with only teaching duties. Compared with developed countries (e.g., <6% in U.S.) [4] and other developing countries that is, average 37% of physicians in Central/Eastern Europe, 29% in Africa, 25% in Central and South America, and 17.5% in Asia were smokers [5], these results reveal the high prevalence of cigarette smoking among Chinese medical professionals.

Studies of smoking behavior have often considered teacher smokers to be one homogeneous group, for example, those reporting smoking at least 100 cigarettes in their lifetime (smokers) [12,14,22], or those reporting smoking at least one in the past 30 days (current smokers) [13]. However, considerable heterogeneity exists among cigarette smokers [23,24,25]. This present study utilized latent class analysis to examine differentiation in smoking behaviors among medical teachers, in order to identify and characterize subgroups with similar patterns of smoking. Our analyses found three latent classes of smoking consumption severity among the present sample. Considerable heterogeneity was found existing among these three subgroups.

The demographic characteristics that separated these groups were gender and age. Specifically, compared to Class I (never smokers and people who did not smoke currently), people with heavier smoking patterns (Class III) were more likely to be older. Similar findings were also replicated by another study that age was positively correlated with smoking prevalence [12]. As aging, smokers may suffer more from the hazard of smoking [26,27], it is important to stop the growing tendency of smoking prevalence with the increase of age. In our study, although 58.3% of Class III wanted to quit smoking, the high dependence score of RRSQ among people with heavier smoking patterns indicated that they would experience more difficulty or discomfort in the absence of cigarettes [19]. In other words, it is more difficult for heavy smokers to quit smoking because of their physical response. Therefore, it is unsurprising that almost half of heavy smokers (48.8%) failed in quitting smoking in the past 12 months. It also indicated that differences of physical dependence on tobacco existed among sub-groups, and high dependence may require more professional help, like Nicotine Replacement Therapy (NRT). Besides, addition and sedative were the most common smoking motive factors for medical teachers in this study, which suggested that medical teachers were more likely to be psychologically addicted to smoking, as well as value smoking as a means to release pressure and anxiety [19,20]. Thus, targeted intervention based on smoking consumption and smoking motive is needed.

Notably, with such high failure rate in tobacco cessation, there were also more than half of respondents (60.9%) who thought quitting smoking was not difficult, though most of them were well informed of smoking-related knowledge. It reveals that Chinese smokers, even medical professionals, may underestimate the addictive nature of tobacco [10], and have unrealistic expectations of how easy quitting will be [28]. Besides, a substantial proportion of participants appeared to be misinformed or unsure regarding the effect of filter, low-tar and low-nicotine cigarettes on reducing the harmful impact of smoking, as well as the age limitation of benefiting from quitting smoking. The insufficiency of smoking-related knowledge among Chinese medical professionals has been reported in many studies [8,9]. As to the importance of scientific knowledge on curbing smoking among medical professionals themselves and to increasing their involvement in smoking cessation services [29], these results emphasize the need for a tobacco cessation training program for medical faculties. Additionally, we found no association between knowledge score and smoking cessation ideation. Consistent with other studies [30,31], it implies that future studies should help to explain the reasons why people continue to smoke despite their knowledge of the risks of smoking.

Although it is claimed that Chinese medical professionals are less involved in tobacco control [32], in this study, most medical teachers appeared to agree that they should set an example by not smoking, and take an active role in tobacco control. It seemed like a good sign because previous studies found that physicians with a more positive attitude toward smoking cessation were more likely to be involved in tobacco control and provide smoking cessation services [5]. However, the univariate analyses of smoking behaviors and attitude showed that, compared with never smokers and non-current smokers, people with higher smoking consumption behaviors were less likely to agree about the responsibilities of medical teachers to refuse smoking and exhort the public to quit smoking. Besides, with such high smoking prevalence, medical teachers did not show the expected healthy image to the public population and future medical professionals (their students). A supportive attitude toward anti-tobacco is in itself not enough for medical professionals to play the leading role sufficiently well in tobacco control and smoking cessation, not to mention to fight with social norms that encourage smoking.

Chinese social norms have been discussed a lot as contributing factors to tobacco use among the Chinese public including medical professional [21,32]. There is a Chinese saying that “a cigarette builds a bridge, while wine builds a road.” [33]. Cigarette sharing is a pervasive Chinese behavior, which serves as a means to show respect and solidify relationships between people in China [34,35,36]. Unsurprisingly, we found that the majority of respondents (62.7%) in this study stated that smoking could improve social communication, especially heavy smokers. Almost half of current smokers (43.4%) reported that if someone offered a cigarette to them, and asked them to smoke together with them, they would be very pleased to accept, as refusing an offered cigarette is viewed as impolite behavior in China [35]. To decouple cigarettes from their social significance requires firm actions at multiple levels including policy implementation, increasing tobacco taxation, mass media campaigns, and public education, where the Chinese government should play the key role. Although the Chinese government has made many efforts to fight tobacco use, it has not yet been enough to curb the burgeoning epidemic. Sustained efforts are still needed.

In the present study, almost two-thirds (63.8%) of current smokers admitted to smoking on campus or in hospital, where students and patients could observe their behavior, which may raise concerns about the efficacy and sincerity of efforts on establishing smoke free schools and hospitals, as in other researches [33]. Fortunately, according to the latest survey conducted by the Chinese Association on Tobacco Control about the smoking-free environment in campus [37], great progress has been made to improve the level of the smoking-free environment of universities in Hunan province. Compared to 2011, the score of the smoking-free environment of universities in Hunan province was increased from 28.53 to 54.72 in 2013 (full score was 100), and the percentages of cigarette butts and smoking sets found indoors were significantly decreased. However, a sustained and comprehensive effort, such as building policies to ban smoking in public places on campus and hospitals, and enhancing monitoring, is still needed to improve and maintain smoke-free settings among universities and hospitals.

There were some limitations for our study. Although the smoking status in Xiaya medical school is typical, caution is needed in generalizing and applying the results to different samples. Differences in covariates associated with the three latent classes were cross-sectional, and thus, future longitudinal studies among this population are needed to give us insight into developments of the outcomes discussed above.

## 5. Conclusions

In conclusion, the smoking prevalence among medical teachers in Changsha City, China remains high. Even though medical teachers were generally well informed about smoking related knowledge, they still may underestimate the difficulty of quitting smoking. Besides, they held a positive opinion toward their leading role in tobacco control, but sociocultural factors can negatively affect their smoking behavior and motivation for smoking cessation. Programs on smoking cessation training are required, but this is in itself not enough. Future study should develop targeted interventions for subgroups of smokers based on smoking consumption. Despite knowledge, attitude and social factors, the physical dependence on tobacco cannot also be ignored. High dependence on tobacco might need clinical help rather than only education. Last but not least, persistent and effective anti-tobacco efforts are needed to achieve the goals of creating smoke-free campuses and hospitals.

## References

[1] World Health Organization WHO Report on the Global Tobacco Epidemic. http://apps.who.int/iris/bitstream/10665/85380/1/9789241505871_eng.pdf?ua=1.

[2] Chinese Center for Disease Control and Prevention China Report on Tobacco Control. http://www.moh.gov.cn/ewebeditor/uploadfile/2013/05/20130531132109426.pdf.

[3] Chinese Ministry of Health China Report on Tobacco Hazard of Health (Summary). http://jkzx.zggs.gov.cn/n2025c24.aspx.

[4] Tong E.K., Strouse R., Hall J., Kovac M., Schroeder S.A. (2010). National survey of U.S. health professionals’ smoking prevalence, cessation practices, and beliefs. Nicotine Tob. Res..

[5] Abdullah A.S., Stillman F.A., Yang L., Luo H., Zhang Z., Samet J.M. (2014). Tobacco use and smoking cessaion practices among physicians in developing countries: A literature review (1987–2010). Int. J. Environ. Res. Public Health.

[6] Andrew P., Michelle S., Robert R. (2009). Physician smoking status, attitudes toward smoking, and cessation advice to patients: An international survey. Patient Educ. Couns..

[7] Bland B. (2009). China tells doctors to quit smoking to set example to patients. BMJ.

[8] Jiang Y., Ong M.K., Tong E.K., Yang Y., Nan Y., Gan Q., Hu T.W. (2007). Chinese physicians and their smoking knowledge, attitudes, and practices. Am. J. Prev. Med..

[9] Yan J., Xiao S., Ouyang D., Jiang D., He C., Yi S. (2008). Smoking behavior, knowledge, attitudes and practice among health care providers in Changsha city, China. Nicotine Tob. Res..

[10] Gao J. (2011). Chinese medical staff’s smoking behaviors and tobacco control stratigies (a review). Chin. J. Urban Rural Enterp. Hyg..

[11] Shi X.L., Li F. (2010). Status of quit smoking among Chinese medical staff. Chin. J. Health Educ..

[12] Fu Y., Liu N., Wang X., Zhu H., Wang Y. (2012). Sample survey of attitude and behavior towards tobacco control in male teachers, doctors and students in a medical college. Chin. Med. Ethics.

[13] Lu Z.X., Wang L.N., Hu Y., Qu Q., Cui Q., Wang F., Wang C.P., Wang R.F. (2012). Cross sectional investigation on smoking prevalence among medical college teachers. Chin. Prev. Med..

[14] Heydari G., Yousefifard M., Hosseini M., Ramezankhani A., Masjedi M.R. (2013). Cigarette smoking, knowledge, attitude and prediction of smoking between male students, teachers and clergymen in Tehran, Iran, 2009. Int. J. Prev. Med..

[15] China Medical Board Tobacco Control. http://www.chinamedicalboard.org/tobacco_control.

[16] You Y., Yin J.B., Zhao L.Q., Deng H., Guan S.Y., Fan X.X., Sun M., Wu Y.Q. (2012). Smoking behavior and attitude among staff of hospitals. J. Prev. Med. Inform..

[17] Patterson F., Lerman C., Kaufmann V.G., Neuner G.A., Audrain-McGovern J. (2004). Cigarette smoking practices among American college students: Review and future directions. J. Amer. Coll. Health.

[18] Warren C.W., Sinha D.N., Lee J., Lea V., Jones N.R. (2011). Tobacco use, exposure to secondhand smoke, and cessation counseling among medical students: Cross-country data from the Global Health Professions Student Survey (GHPSS), 2005–2008. BMC Public Health.

[19] Russell M.A.H., Peto J., Patel U.A. (1974). The classification of smoking by factorial structure of motives. J. Roy. Statist. Soc. Ser. A.

[20] Hao W., Young D. (1988). Effect of clonidine on cigarette cessation and in the alleviation of withdrawal symptoms. Brit. J. Addict..

[21] Zhang J., Ou J.X., Bai C.X. (2011). Tobacco smoking in China: Prevalence, disease burden, challenges and future strategies. Respirology.

[22] Feng G.Z., Jiang Y., Li X., Yang Y., Wu X. (2009). Survey on smoking-related knowledge, attitudes and behavior of teachers across China. Chin. J. Prev. Control Chronic Dis..

[23] Furberg H., Sullivan P.F., Maes H., Prescott C.A., Lerman C., Bulik C., Kendler K.S. (2005). The types of regular cigarette smokers: A latent class analysis. Nicotine Tob. Res..

[24] Sutfin E.L., Reboussin B.A., McCoy T.P., Wolfson M. (2009). Are college student smokers really a homogeneous group? A latent class analysis of college student smokers. Nicotine Tob. Res..

[25] Chen X., Li X., Stanton B., Mao R., Sun Z., Zhang H., Qu M., Wang J., Thomas R. (2004). Patterns of cigarette smoking among students from 19 colleges and universities in Jiangsu Province, China: A latent class analysis. Drug Alcohol Dependence.

[26] Si X.L., Wang A.F., Wang Z.L. (2014). The relationship between chronic diseases and lifestyle among community old adults: Based on an empirical investigation among thirteen cities in Jiangsu province. Chin. J. Gerontol..

[27] Wang K.S., Wang L., Zheng S., Wu L.Y. (2013). Associations of smoking status and serious psychological distress with chronic obstructive pulmonary disease. Int. J. High Risk Behav. Addict..

[28] Li L., Feng G., Jiang Y., Yong H.H., Borland R., Fong G.T. (2011). Prospective predictors of quitting behaviours among adult smokers in six cities in China: Findings from the International Tobacco Control (ITC) China Survey. Addiction.

[29] Huang C., Guo C., Yu S., Feng Y., Song J., Eriksen M., Redmon P., Koplan J. (2013). Smoking behaviors and cessation services among male physicians in China: Evidence from a structural equation model. Tob. Control.

[30] Demaio A.R., Nehme J., Otgontuya D., Meyrowitsch D.W., Enkhtuya P. (2014). Tobacco smoking in Mongolia: Findings of a national knowledge, attitudes and practices study. BMC Public Health.

[31] Sidani J.E., Shensa A., Barnett T.E., Cook R.L., Primack B.A. (2014). Knowledge, attitudes, and normative beliefs as predictors of Hookah smoking initiation: A longitudinal study of university students. Nicotine Tob. Res..

[32] Abdullah A.S., Qiming F., Pun V., Stillman F.A., Samet J.M. (2013). A review of tobacco smoking and smoking cessation practices among physicians in China: 1987–2010. Tob. Control.

[33] Ma S.J., Wang J.F., Mei C.Z., Xu X.F., Yang G.H. (2007). Passive smoking in China: Contributing factors and areas for future interventions. Biomed. Environ. Sci..

[34] Hu M., Rich Z.C., Luo D., Xiao S. (2012). Cigarette sharing and gifting in rural China: A focus group study. Nicotine Tob. Res..

[35] Ceraso M., McElroy J.A., Kuang X., Vila P.M., Du X., Lu L., Ren H., Qian N., Jorenby D.E., Fiore M.C. (2009). Smoking, barriers to quitting, and smoking-related knowledge, attitudes, and patient practices among male physicians in China. Prev. Chronic. Dis..

[36] Xiao S., Kohrman M. (2008). Anthropology in China’s health promotion and tobacco. Lancet.

[37] Chinese Association on Tobacco Control National Undercover Assessment on Smoke-Free Environment in Universities and Colleges. http://www.tcrc.org.cn/html/zy/cbw/jc/2640.html.

